# Hypoxically Induced Nitric Oxide: Potential Role as a Vasodilator in *Mytilus edulis* Gills

**DOI:** 10.3389/fphys.2018.01709

**Published:** 2019-03-05

**Authors:** Paula Mariela González, Iara Rocchetta, Doris Abele, Georgina A. Rivera-Ingraham

**Affiliations:** ^1^Facultad de Farmacia y Bioquímica, Universidad de Buenos Aires, Fisicoquímica, Buenos Aires, Argentina; ^2^Instituto de Bioquímica y Medicina Molecular (IBIMOL), CONICET-Universidad de Buenos Aires, Buenos Aires, Argentina; ^3^Laboratorio de Ecotoxicología Acuática, INIBIOMA, CONICET-COMAHUE, Neuquén, Argentina; ^4^Department of Biosciences, Alfred Wegener Institute Helmholtz Centre for Polar and Marine Research, Bremerhaven, Germany; ^5^Laboratoire Environnement de Petit Saut, Hydreco-Guyane, Kourou, French Guiana

**Keywords:** blood vessel opening, blue mussels, hypoxia, mitochondria, nitric oxide

## Abstract

Intertidal *Mytilus edulis* experience rapid transgression to hypoxia when they close their valves during low tide. This induces a physiological stress response aiming to stabilize tissue perfusion against declining oxygen partial pressure in shell water. We hypothesized that nitric oxide (NO) accumulation supports blood vessel opening in hypoxia and used live imaging techniques to measure NO and superoxide anion (${\text{O}}_{2}^{∙-}$) formation in hypoxia-exposed gill filaments. Thirty minutes of moderate (7 kPa pO_2_) and severe hypoxia (1 kPa pO_2_) caused 1.6- and 2.4-fold increase, respectively, of NO accumulation in the endothelial muscle cells of the hemolymphatic vessels of the gill filaments. This led to a dilatation of blood vessel diameter by 43% (7 kPa) and 56% (1 kPa), which facilitates blood flow. Experiments in which we applied the chemical NO-donor Spermine NONOate (concentrations ranging from 1 to 6 mM) under normoxic conditions corroborate the dilatational effect of NO on the blood vessel. The formation of ${\text{O}}_{2}^{∙-}$ within the filament epithelial cells increased 1.5 (7 kPa) and 2-fold (1 kPa) upon treatment. Biochemical analysis of mitochondrial electron transport complexes in hypoxia-exposed gill tissue indicates decreased activity of complexes I and III in both hypoxic conditions; whereas complex IV (cytochrome-c oxidase) activity increased at 7 kPa and decreased at 1 kPa compared to normoxic exposure conditions. This corresponds to the pattern of pO_2_-dependent gill respiration rates recorded in *ex-vivo* experiments. Severe hypoxia (1 kPa) appears to have a stabilizing effect on NO accumulation in gill cells, since less O_2_ is available for NO oxidation to nitrite/nitrate. Hypoxia thus supports the NO dependent inhibition of complex IV activity, a mechanism that could fine tune mitochondrial respiration to the local O_2_ availability in a tissue. Our study highlights a basal function of NO in improving perfusion of hypoxic invertebrate tissues, which could be a key mechanism of tolerance toward environmental O_2_ variations.

## Introduction

*Mytilus edulis*, the blue mussel, is a bank forming species that colonizes intertidal and subtidal habitats. It belongs to the group of outstandingly hypoxia and anoxia tolerant marine invertebrates, endowed with specialized “anaerobic mitochondria” that can alternate between the use of oxygen (O_2_) and of endogenous fumarate as electron acceptor for anaerobic ATP production (Tielens et al., 2002). The extent of hypoxia and of anoxia tolerance, however, varies with individual environmental adaptation. In intertidal environments blue mussels experience a reduction of shell water O_2_ partial pressure (pO_2_) to hypoxic or even anoxic levels during low tides caused by intermittent valve closure that prevents desiccation (Bayne et al., 1976; for review see Abele et al., 2017). Transplant experiments with subtidal and intertidal mussels between both habitats demonstrated hypoxic tolerance to be higher in intertidal than subtidal mussels, but also to be enhanced within weeks after transplantation and adaptation to the intertidal (Altieri, 2006). Hence hypoxia tolerance in blue mussels has an acquired and adaptive component modulating the evolutionary trait. Depending on length and intensity, anoxic exposure can cause cellular stress, including oxidative stress when cells are re-oxidized during valve opening (Rivera-Ingraham et al., 2013b).

**Figure 1:**
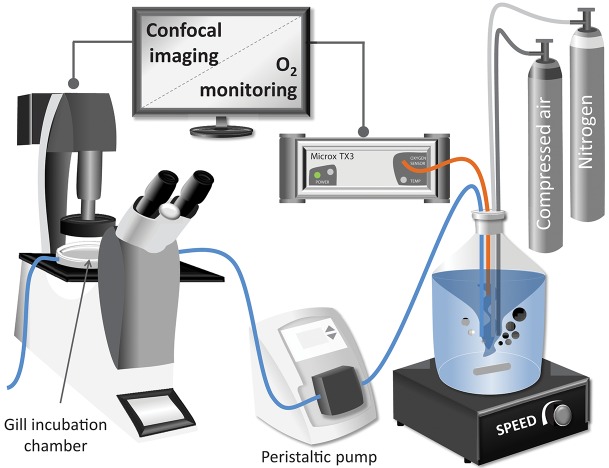
Schematic representation of the experimental setup used for the confocal analysis of excised mussel gills.

Gills are the main organs of respiration in bivalves and, contrary to other diffusive surfaces such as mantle, can functionally stabilize the rates of whole animal respiration against fluctuant environmental O_2_ concentrations. Enhanced ciliary (ventilation) and heart beat rates (perfusion) (Bayne, 1971), combined with a widening of the inter-lamellar blood vessel, caused by contraction of the muscles in the inter-lamellar connections (Aiello and Guideri, 1965, named “intracellular junctions” in Figure 1), are central mechanisms by which *Mytilus* can stabilize respiration rates against declining O_2_ availability. Using freshly excised gills, we demonstrated a distinct pattern of increasing respiration rate below ~9.5 kPa (critical pO_2_ 1) (pc1) in support of faster ciliary beating at lower pO_2_, before onset of oxyconformity at ~6.5 kPa (pc2) (i.e., 35–40% of the normoxic level, Rivera-Ingraham et al., 2013b). It is an open question how this complex response pattern of O_2_ turnover in mussel gill mitochondria is regulated. Cytochrome c oxidase (CytOx) is generally accepted to be the rate limiting factor of mitochondrial O_2_ turnover, but its affinity for O_2_ would need to change dramatically in the O_2_ range above 7 kPa to achieve the activity pattern observed in our previous study. Alternatively, another O_2_ related molecule could be functioning as a mediator between pO_2_ levels and CytOx - O_2_ affinity.

Nitric oxide (NO) is a reactive nitrogen species (RNS) that plays an important role as cellular mediator, specifically with respect to its interaction with O_2_ at the CytOx reactive center (Taylor and Moncada, 2010). Intracellular formation of NO is almost exclusively catalyzed by NO synthases, a group of heme-based monooxygenases present in different tissues of marine and freshwater molluscs, including the central nervous system (Moroz et al., 1996), molluscan hemocytes cells (Conte and Ottaviani, 1995; Tafalla et al., 2003; Palumbo, 2005); and bivalve digestive glands (González et al., 2008; González and Puntarulo, 2011). More recent investigations into microbial biofilms on internal surfaces, external structures (shells), and in gut contents of marine and freshwater molluscs highlight nitrification/ denitrification processes of associated facultative anaerobic bacteria to be another potential source of NO and nitrous oxide (N_2_O) in marine invertebrates (Heisterkamp et al., 2010; Svenningsen et al., 2012; Stief, 2013). Especially under near anaerobic conditions N_2_O and NO form as products of nitrite (${\text{NO}}_{2}^{-}$) reduction, similar to denitrification processes in anoxic sediment layers (Anderson and Levine, 1986; Stief, 2013). Whether NO produced by microbial denitrification in molluscan shell water, or the NO produced inside the cells by NO synthase activity can have an effect on gills or hemocyte cells and respiratory activities, is so far unexplored.

At least for mammalian cells the interactions between NO and respiration rates are sufficiently clear. Mammalian CytOx has a higher affinity for NO than for O_2_ and catalyzes its oxidation to ${\text{NO}}_{2}^{-}$ at normoxic cellular pO_2_ (note that “normoxic cellular pO_2_” is much lower than 21 kPa aerial partial pressure in bivalve tissues, and even lower in mammalian cells). At high pO_2_, this oxidation occurs in a manner that is non-competitive to O_2_, which means that NO oxidation and respiration, two O_2_ consuming processes, proceed simultaneously. As O_2_ diminishes in hypoxia, the CytOx reactive center becomes reduced, which causes NO binding at the catalytic site for the O_2_ reduction (the heme a_3_ in its ferrous state). This abrogates NO oxidation to ${\text{NO}}_{2}^{-}$ and stabilizes intracellular NO levels, which will further reduce and eventually fully inhibit CytOx catalytic activity (for a detailed description of the biochemical mechanism underlying the interaction between NO and CytOx see Taylor and Moncada (2010) and references cited therein. Thus, NO can have a mediator function in mammalian cells, diminishing CytOx catalytic activity in an O_2_ dependent manner at the onset of hypoxia. The physiological effect of the curtailed O_2_ consumption is a better diffusive distribution of O_2_ across hypoxia sensitive mammalian tissues, in which the peripheral cells would have better access than the cells in central tissue regions (Poderoso et al., 1996).

Bivalves have open circulatory systems and O_2_ distribution occurs over the hemolymph that, in most bivalves including Mytilides, is void of O_2_ binding respiratory proteins. Big hemolymphatic vessels run through the gill branches and filaments and also connect the heart with the major tissues, foot, mantle/gonads, and digestive tract for O_2_ supply. Heart beat is controlled by the inspired pO_2_ (and not the pCO_2_) detected by peripheral O_2_ sensors within the inhalant siphon (Abele et al., 2017).

A suitable model to mechanistically study O_2_ transport and the biochemical and functional responses of cells and their mitochondria to diminishing O_2_ levels *in vitro* is the intact gill, immediately after its removal from the living mussel. In our previous papers we used live imaging techniques in combination with fluorescent dyes to measure the response of the gills to O_2_ deprivation and reoxygenation in terms of reactive species formation and oxidative damage accumulation (Rivera-Ingraham et al., 2013b). We also investigated the compartmentalization of the different reactive O_2_ species (ROS) to better understand their diverse functions in the gills. It resulted that DAF-2DA fluorescence (NO) and dichlorofluorescein diacetate (DCF) staining (ROS and RNS) are compartmentalized in the endothelial muscle cells around the hemolymphatic sinus of the filaments, and additionally stain hemocyte cells within the vessel lumen (especially DCFH, see Rivera-Ingraham et al., 2016). Contrary the ${\text{O}}_{2}^{∙-}$ sensitive dye dihydroethidium (DHE) stained the palisade cells of the gills and here the outer ciliated and mitochondria rich areas fluoresced most strongly. Especially the distinctive staining of the longitudinal endothelial muscle cells around the blood vessel by the NO sensitive fluorophore DAF-2D suggested that NO could be a messenger molecule involved in the hypoxic adjustment of the hemolymphatic vessel lumen to regulate blood pressure under hypoxic conditions. Hence in the present paper we investigated the hypoxic NO accumulation in endothelial muscle cells and, in parallel, determined blood vessel diameter in the filaments.

To better understand potential effects of NO accumulation on cellular and mitochondrial processes in the gills, we compared the effects of natural and hypoxic NO accumulation to experimental addition of the NO donor (SpermineNONOate, SpNONOate) on gill respiration, mitochondrial membrane potential and ROS formation. Inhibitory effects of hypoxia and of externally added NO on mitochondrial respiratory chain components (electron transport system, ETS, complexes I and III and CytOx) were tested directly *in vitro* using gill homogenates. The overall aim of our study was to understand whether NO accumulation in hypoxic gills can lead to modifications of mitochondrial respiratory complex activities and gill perfusion (vessel diameter) in a hypoxia tolerant and partially oxyconforming marine bivalve.

## Materials and Methods

### Animal Collection and Maintenance

*M. edulis* were collected at the Island of Sylt in the North Sea, Germany (55° 01′ 323 N and 008° 26′ 430 E) during autumn 2016 after the reproductive season (spring and summer). With a mean shell length of 39.5 ± 0.3 mm all mussels were beyond the period of strongest growth for North Sea populations, and were considered to represent young adults (Sukhotin et al., 2006). No distinctions were made regarding gender. The animals were transferred to the laboratory (Alfred-Wegener-Institute Helmholtz-Zentrum für Polar- und Meeresforschung, AWI), cleaned from epibiontic growth, and kept completely submerged in two aquaria with fully aerated (>99 % air saturation) natural seawater of 32.3 %0 at 10°C. Mussels were allowed to acclimate to the aquarium conditions for 3 weeks prior to the experiments and were fed live phytoplankton using PhytoMaxx Live Plankton Concentrate (NYOS Aquatics GmbH, Korntal-Muenchingen, Germany) (500 x 10^6^ cell·mL^−1^) once a week. During feeding water circulation in the aquaria was stopped for 4 h. Mussels were fasted for 48 h before experimentation to avoid possible interference of nutrition-induced increase in metabolic rates. Water quality was monitored weekly for ammonium and nitrate levels using Nanocolor® Tube Tests (Macherey-Nagel GmbH & Co. KG, Düren, Germany). Water in the aquaria was changed when ammonium values exceeded 0.4 mg·L^−1^ or when nitrate values exceeded 0.2 mg·L^−1^.

### Experimentation With Live Tissues

Respiration rates and live imaging of physiological parameters were carried out *ex-vivo*, using freshly excised mussel gill pieces or isolated filaments. Mussels for these experiments were obtained directly from the acclimation aquaria and sacrificed on ice. The entire gill was removed and kept in 15 mM Na-HEPES and 0.5 mM glucose (NH-FSW) for experimentation.

Experimental exposure *ex-vivo* included different pO_2_ levels: normoxia (21 kPa pO_2_), moderate (7 kPa pO_2_, coinciding with the critical pO_2_ for *M. edulis* excised gills (Rivera-Ingraham et al., 2013b), and severe hypoxia of 1 kPa pO_2_ to mimic conditions in shell water of naturally hypoxia-exposed or shell-closed mussels. To test for the effect of NO on the same functions, freshly excised gill tissue was exposed to different concentrations of the NO donor SpNONOate (Sigma S-150) under normoxic conditions in an open chamber (i.e., pO_2_ conditions were maintained constant between 18 and 20 kPa throughout these experiments) used for the live imaging experiments. Measurements of respiration rates were conducted in a closed system (see section Respirometry of excised gill pieces exposed to Spermine NONOate) in which the gill pieces were maintained with SpNONOate while pO_2_ decreased in the chamber, so that the results could be evaluated for different ranges of mild and severe hypoxic exposure.

SpNONOate was chosen as NO donor because of its long half-life of 230 min between 22° and 25°C (Sinha, 2011). Previous measurements in our laboratory demonstrated a slight pO_2_ dependence of NO formation/accumulation by SpNONOate in that a 6 mM SpNONOate generated approximately 50 nM · min^−1^ at 16 kPa (Julia Strahl, pers. comm).

#### Respirometry of Excised Gill Pieces Exposed to Spermine NONOate

The wells of a 96-well Nunclon plastic microtiter plate (NunclonTM Nalge Nunc, Denmark) served as respiration chambers. Wells with a volume of 0.33 mL and a diameter of 8 mm were equipped with O_2_ sensor spots. A 4-channel fiber-optical O_2_ meter (Oxy-4) and noninvasive O_2_ sensors (SP-PSt3-NAU-D5-YOP, Precision Sensing GmBH, Regensburg, Germany) were used after daily calibration following the manufacturer's description. Spots were glued to the bottom of the wells using silicon paste. Animals were dissected, and freshly excised gills were divided into two approximately equal sections. Since the amount of tissue in a respiration chamber can have an effect on the O_2_ consumption measurements (Van Winkle, 1968), approximately the same amount of tissue (10–15 mg fresh weight, FW) was used for each animal. Following the measurement, the exact FW of each gill piece was determined after blotting it dry on tissue paper. Two gill pieces per animal were placed individually in respiration wells: one containing sterile NH-FSW, and the other one containing NH-FSW supplemented with either 1, 3, or 6 mM SpNONOate. Wells were filled completely with normoxic medium and sealed (to avoid the formation of air bubbles) as described in Rivera-Ingraham et al. (2013b). All measurements started in fully oxygenated medium and respiration was recorded as function of declining pO_2_ over time. Measurements were conducted at room temperature (20°C). Data were recorded at 5 s intervals until complete anoxia was reached in a well-chamber, or until gills stopped breathing. Measuring time was always less than 5 h.

Respiration data over the whole range of pO_2_ from normoxia to anoxia were divided into three pO_2_ range sections: (i) normoxia from 21-15 kPa, (ii) range around the critical pO_2_ previously determined for the same *M. edulis* population (Acevedo et al., 1993; Rivera-Ingraham et al., 2013b): 8.5–5 kPa and including our experimental treatment of moderate hypoxia, and (iii) severe hypoxia 4-0 kPa representative of the experimental treatment at 1 kPa. Data for each pO_2_ interval were fitted by linear regression and the corresponding slope was used to calculate gill respiration rate as nmol O_2_ · min^−1^ · mg^−1^ FW. For each individual, gill respiration rate was calculated for treatments with and without the presence of SpNONOate in the respiration medium and for the respective pO_2_ ranges. Results are given as respiration rates for each range and the percentage of inhibition by SpNONOate for each individual.

#### Fluorometric Analyses of Nitric Oxide and Superoxide Anion Formation, and Mitochondrial Membrane Potential

Live imaging of NO and ${\text{O}}_{2}^{∙-}$ formation, as well as the changes of the mitochondrial membrane potential (Δψ_m_) in live gill filaments under different treatment conditions was conducted using a Leica TCS SP5II confocal microscope (Leica Microsystems CMS GmbH, Wetzlar, Germany) equipped with a multiphoton laser (MaiTai-DeepSee, Spectra-Physics, Newport Corp.). A 40X optical objective was used with a resolution of 500 × 500 pixels. Quantification of fluorescence intensity was done with Leica LAS AF-TCS-SPS Lite software (Leica Microsystems CMS GmbH 2011, Version 2.6.0).

Freshly excised demibranch pieces of mussel gills were loaded with the corresponding fluorophore at room temperature (20°C) under normoxic (loading) conditions and for the time indicated in Table 1. Loading and measuring medium was in all cases NH-FSW. Chemical reaction mechanisms, concentrations of fluorophores, incubation times, as well as the conditions of visualization are summarized in Table 1.

**Table 1:** Analysis conditions for each of the dyes used during the study.

**Dye**	**Mechanism of function**	**N per treatment**	**Final Concentration (μM)**	**Incubation time (min)**	**Excitation**		**Emission**		**Calculation**
					**λ_1_ (nm)**	**λ_2_ (nm)**	**PMT1 (nm)**	**PMT2 (nm)**	
DAF-2DA (in DMSO)	DAF-2 is formed by intracellular hydrolyzation of its ester bonds by esterases. It remains essentially non-fluorescent until it reacts with nitrosonium cation (forming the fluorescent DAF-2T) and such fluorescence increases in a NO-dependent manner.	7	20	30	488	–	505–525	–	Average intensity
MitoSOX (in DMSO)	Dye targeting mitochondria where it is readily oxidized by ${\text{O}}_{2}^{∙-}$ the oxidized product becomes fluorescent upon binding to nucleic acids.	8	5	30	514	–	560–600	–	Average intensity
JC-10 (in DMSO)	Green fluorescent probe which exists as a monomer at low Δψm. With high Δψm values JC-10 aggregates shows a red fluorescence.	6	10	30	488	488	500–550	560–600	Ratio PMT1/PMT2

*PMT, Photomultiplier tube*.

After loading of the fluorophore, gill filaments were placed and fixed in the confocal chamber containing 2 mL of NH-FSH with a pO_2_ at normoxic conditions. The confocal chamber (Bachofer, with a diameter of 5 cm) was perfused with NH-FSW medium at 1.77 mL · min^−1^ for 30 min of experimental treatment (see Figure 1 for experimental setup). The pO_2_ in the incubation medium was controlled in the reservoir. Room temperature was maintained between 18 and 20°C throughout the measurement.

For each gill sample, an area comprising one or two filaments (area of analysis, AA) was chosen far from the dissection border and showing no sign of manipulation damage of the gill structures. Only this area was analyzed throughout the experiment (lasting 30 min). All image adjustments, including fluorescence gain and selection of the AA were accomplished within a maximum of 3 min of pre-incubation in the chamber before starting the measurement (normoxic conditions, serving as control and expressed as 0 min in figures). The system was then either maintained under normoxic conditions, changed to moderate (7 kPa pO_2_), or severe hypoxia (1 kPa pO_2_), or treated with either 3 or 6 mM SpNONOate at normoxic conditions. For the hypoxically incubated gill, the medium in the flow-through system was replaced by hypoxic medium immediately after the initial confocal measurement under control conditions. To minimize photobleaching, for each AA one single image was taken at each time point (0, 10, 20, and 30 min, 4 images in total per AA). Only one AA was selected per gill piece and only one gill piece was used per mussel. A minimum of 4 mussels were used per fluorophore. The pO_2_ of the hypoxic medium was constantly monitored directly in the reservoir and pO_2_ was also measured in the perfusion chamber at the beginning and at the end of the experiment using a calibrated O_2_ meter Microx TX3 (Presens GmbH, program Oxy View TX3-V6.02) equipped with a needle type O_2_ micro-sensor NTH-PSt 1. For normoxic exposures, the Bachofer chamber was maintained open. For hypoxic conditions the chamber was closed with a coverslip and perfused with hypoxic medium.

**Figure 2:**
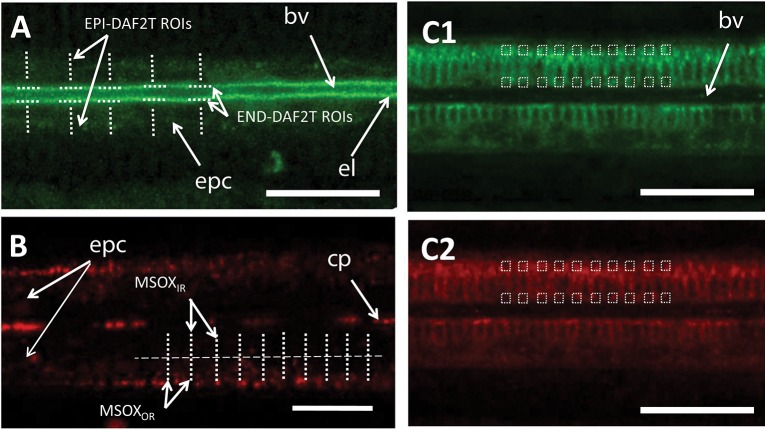
Schematic representation of the number and type of regions of interest used for the image analyses of confocal experiments using: **(A)** DAF-2DA, **(B)** MitoSOX, and **(C)** JC-10 in the **(C1)** green and **(C2)** red spectrum ranges. Scale bars: 20 μm. bv, blood vessel; cp, circulating particles; epc, epithelial cells; el, endothelial lining surrounding the blood vessel. Dotted lines indicate regions of interest.

Nitric oxide formation in gill filaments was visualized using DAF-2DA (Sigma D225). For each of the pictures taken, a total of 10 regions of interest (ROIs) were defined on epithelial cells, perpendicularly to the longitudinal axis of the gill filament (Figure 2A). For each of these ROIs the average fluorescence intensity was calculated (EPI-DAF2T). Additionally, another 10 ROIs were defined on the endothelial cells, in this case parallel to the longitudinal axis of the filament and the average fluorescence intensity was equally calculated (END-DAF2T). Since absolute DAF-2DA loading differs between gills, EPI-DAF2T values were used to obtain a ratio (END-DAF2T: EPI-DAF2T) (called fluorescence ratio from now on) for each picture to allow comparisons between gill pieces.

The formation of ${\text{O}}_{2}^{∙-}$ was assessed by incubating a minimum of 4 gills pieces per time point (one gill piece per mussel) with the fluorescent dye MitoSOX (Invitrogen M36008). For each image, 10 ROIs were defined perpendicularly to the longitudinal axis of the gill filament (Figure 2B). The region of the blood vessel lumen was not considered in this analysis to avoid interference from the high fluorescence emitted by hemocyte cells. Given that the maximum MitoSOX fluorescence was in all cases located in the outer region of epithelial cells (Rivera-Ingraham et al., 2016), each of these ROIs was subdivided in two equally sized regions at higher image resolution (outer and inner regions and in all cases excluding the vessel lumen as in Rivera-Ingraham et al., 2016). Two values were then calculated**:** (i) the average fluorescence intensity of the ROI inner region (MSOX_IR_) and (ii) the average fluorescence intensity of the ROI outer region (MSOX_OR_). Then, the MitoSOX fluorescence for each sample was calculated as MSOX_OR_-MSOX_IR._

The fluorescence probe JC-10 (Enzo Life Sciences ENZ-52305) was used to observe the differences in the Δψm in both epithelial and endothelial gill cells. In each image fluorescence analysis was done using square ROIs (approximate diameter of 1.5 μm) (Figure 2C): (i) 10 ROIs were evenly distributed in the region of the epithelial cells of the filament and (ii) 10 in the endothelial cells. Mean intensity values were recorded for each square for both the green (JC10ENDgreen and JC10EPIgreen for endothelial and epithelial cells, respectively; Figure 2C1) and the red channels (JC10ENDred and JC10EPIred for endothelial and epithelial cells, respectively; Figure 2C2). The ratio of red fluorescence over green fluorescence was then calculated separately for both endothelia and epithelial cells as a measure of changes in Δψm using Leica LAS AF Lite software (Leica, Microsystems CMS GmbH 2011).

### Measurements of Blood Vessel Diameters

Using the same 4-8 bivalves as for DAF-2DA measurements, blood vessel diameter was measured in freshly excised gills under normoxic (control) and treatment conditions. Treatments were conducted in the perfusion chamber of the confocal microscope, using the same set-up described in the next section: Mitochondrial respiratory complex assays in gill homogenates. During the incubations, the gills were maintained at room temperature (18°C) and were exposed under the respective gaseous atmosphere in NH-FSH for each time point (0, 10, 20, and 30 min). A 40X optical objective was used for imaging and 13 vertical sections (Z-stack images) were taken at 1.4 μm steps. For each time point, the image from the Z-stack showing the widest blood vessel diameter was selected for quantification. In order to measure the overall width of the blood vessel for each image, 10 width measurements were conducted of the inner vessel lumen just up to the limits of the surrounding endothelial cells. Images were taken and analyzed along the gill filament. Given that the blood vessel diameter is not consistent along the filament, the largest diameter determined in each image was used for quantification, using the software routines to measure distances on imaged structures.

### Mitochondrial Respiratory Complex Assays in Gill Homogenates

Measurements of respiratory complex activities were conducted on frozen gill pieces that were homogenized and incubated under experimental pO_2_ conditions or in the presence of SpNONOate for 3 h before the activity measurement. Mitochondrial respiratory complex I, III, and IV assays were performed in the incubated homogenates *in-vitro* no later than 48 h after freezing the gills.

Each frozen gill was cut into equal pieces that were weighed to the nearest 0.1 mg. One piece of each individual gill was used for one out of five experimental pO_2_ and NO treatments (normoxia: 21 kPa, hypoxia: 7 kPa, severe hypoxia: 1 kPa, normoxia + 3 mM SpNONOate and normoxia + 6 mM SpNONOate) to enable direct comparability between treatments for each individual. Each gill piece was homogenized in a medium containing 20 mM Tris (hydroxymethyl) aminomethane supplemented with 1 mM EDTA, 0.1% Tween 20, and SpNONOate (for NO treatments only) at 7.4 pH. Adjustment of treatment conditions (pO_2_ and addition of NO donor) were established by equilibrating the homogenization buffer with air (for normoxic conditions) or a mixture of air and nitrogen (N_2_) (for hypoxia). To control buffer-pO_2_, the respirometer described in the respirometry description was used. For hypoxic exposures, homogenization and subsequent incubation were carried out in a hypoxic atmosphere (achieved by the same air and N_2_ mixture used for equilibrating the homogenization buffer). Once the desired pO_2_ was reached, the required volume (i.e., 1/6: w/v) was transferred under hypoxic atmosphere (for the hypoxic treatments) to a 1.5 mL tube containing the sample. Final homogenate volumes ranged between 62 and 230 μL. Homogenization was done at 4°C using a Precellys Homogenizer 24 (2 cycles × 15 s 5,000 rotations and 15 s break) equipped with a cryolysis advanced temperature controller (Bertin Technologies, Montigny-le-Bretonneux, France). The preparation of the microplate was also carried out under normoxic/hypoxic atmosphere, as required. The final activity measurement was conducted at normoxic conditions and at room temperature (20°C) in a TriStar microplate reader (Berthold Technologies, Bad Wildbad, Germany).

Activity of the ETS was measured according to Châtelain (2008) at λ = 485 nm with ε = 15.9 mM^−1^ · cm^−1^. Each well of a 96 well microplate contained: 117 μL of the measuring buffer (100 mM imidazole at 8.0 pH), 20 μL of 0.1 M sodium azide, 49 μL of 7.9 mM iodonitrotetrazolium chloride (INT, Sigma I-8377) to which 4 μL of the homogenized sample were added. All solute ions were equilibrated at the three experimental pO_2_ conditions (21, 7, 1 kPa). A blank signal was allowed to stabilize for a maximum time of 10 min (pre-run), and the reaction was triggered by injection of 10 μL of 8 mM NADH into each well. The final reaction volume per well was 200 μL. The increase in absorbance was recorded at intervals of 30 s for an entire duration of 10 min (main run). Plates were shaken between measurements to avoid precipitation of INT using the ellipsoidal shaker function of the instrument at medium velocity in backward and forward mode. The ETS activity in the extract was calculated by subtracting the slope of the pre-run from the slope of the main run (abs · min^−1^). Results are expressed as U · g FW^−1^.

CytOx activity was measured after Moyes et al. (1997) at λ = 550 nm with ε = 19.1 · mM^−1^ · cm^−1^. The measurement of CytOx is based on the oxidation of reduced cytochrome c by CytOx. A 5 min pre-run was conducted using 170 μL of the measuring buffer (20 mM Tris HCl with 0.5% Tween 20, 8.0 pH) and 20 μL of the homogenate. The reaction was started by the addition of 10 μL of reduced cytochrome c (200 μL in total per well). The reduced cytochrome c solution was prepared by dissolving 100 mg of cytochrome c in 4 mL of in N_2_-bubbled reduction buffer (20 mM Tris HCl, 8.0 pH) and adding a small amount of sodium dithionite under a N_2_ atmosphere. The reduced cytochrome c was passed over a Sephadex G-25 column using 10 mL of anoxic reduction buffer. Absorbance decrease reflecting the oxidation of cytochrome c was measured over 5 min (main run) at 15 s measuring interval. The samples were shaken between each measurement and activity was calculated by subtracting the slope of the pre-run from the slope of the main run (abs · min^−1^). CytOx activity is expressed as U · g FW^−1^.

###  Statistical Analyses

All data was tested for normality (Kolmogorov-Smirnov test) and homocedasticity (Levene test). A one-way ANOVA was carried out if the requirements for parametric analysis were met, followed by a Student-Newman-Keuls *post-hoc* test. For the rest of cases, a Kruskal-Wallis test was conducted, followed by U-Mann Whitney pairwise comparisons. The level of significance was *p* < 0.05 if not otherwise indicated.

## Results

### Respiration Experiments

**Figure 3:**
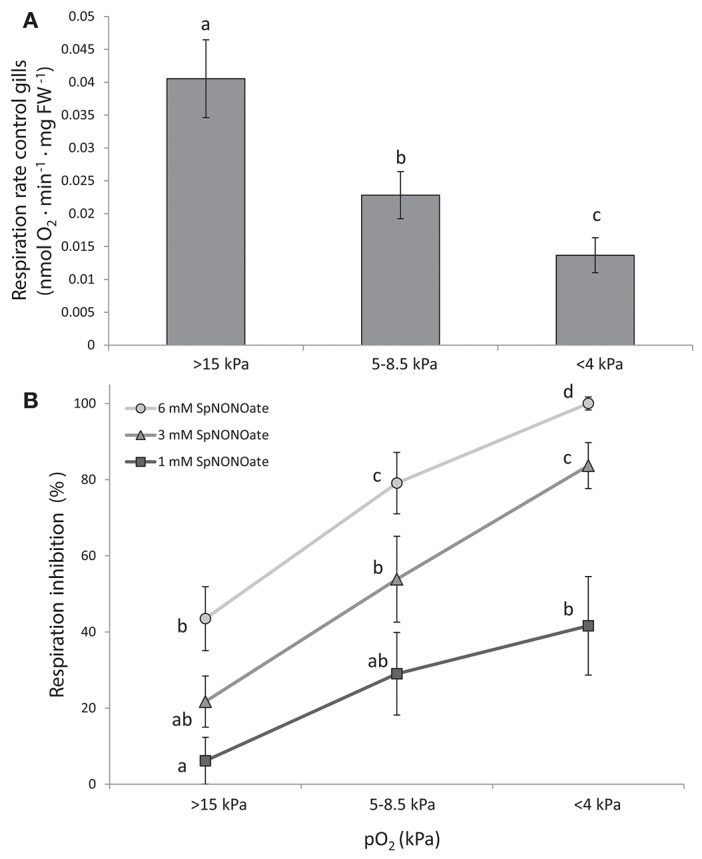
Respiration rates of *Mytilus edulis* excised gills at different O_2_ partial pressures under: **(A)** control conditions and **(B)** different concentrations of Spermine NONOate. Results in **(B)** are expressed as the percentage of inhibition that Spermine NONOate induces in gill respiration compared to the values obtained in undisturbed gill tissues of the same individual. Letters indicate significant differences between mean values based on Kruskal-Wallis test followed by U Mann-Whitney pairwise comparisons.

Respiration rates of *M. edulis* gill pieces significantly decreased in a pO_2_-dependent (oxyconforming) manner (Figure 3A) (*K* = 16.285; *p* < 0.001, *n* = 24) with moderate (5–8.5 kPa) and acute (<4 kPa) hypoxia causing a 1.8- and 3-fold reduction over normoxic conditions, respectively. Addition of SpNONOate reduced respiration rates in a concentration dependent manner (Figure 3B, *n* = 8). At each SpNONOate concentrations, the inhibitory effect increased at lower pO_2_ to the point that gills exposed to the highest NO donor amounts (6 mM) showed complete respiratory shut down below 4 kPa.

### Response of Isolated Gills to Hypoxia and Experimental Nitric Oxide Exposure (*ex vivo*)

**Figure 4:**
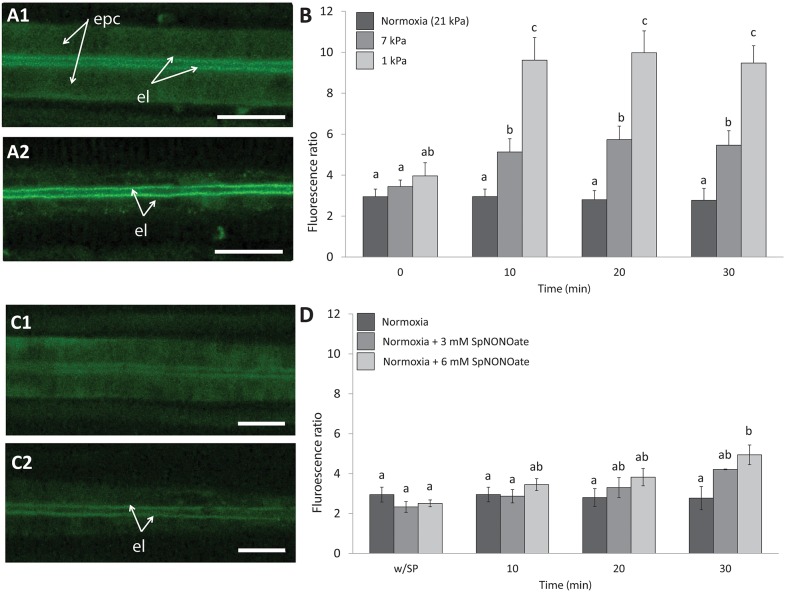
Nitric oxide formation as shown by DAF-2T fluorescence in *Mytilus edulis* gill tissues over time when exposed to different: **(A,B)** Degrees of hypoxia or **(C,D)** Concentrations of Spermine NONOate. **(A)** Representative images of the gills of the same individual with DAF, **(A1)** taken under control (normoxic) conditions and **(A2)** under severe hypoxia 1 kPa for 30 min. **(C)** Representative images of the gills of the same individual under **(C1)** normoxia and **(C2)** incubated with 6 mM Spermine NONOate for 30 min. Quantitative data are shown in **(B,D)** and values were expressed as the average fluorescence intensity of END-DAF2T: EPI-DAF2T. Letters indicate significant differences between mean values based on Kruskal-Wallis test followed by U Mann-Whitney pairwise comparisons. Scale bars: 20 μm; epc, epithelial cells; el, endothelial lining surrounding the blood vessel.

Nitric oxide formation (DAF-2T fluorescence) was mainly detectable in the endothelial cells surrounding the blood vessel and less intense in the epithelial cells in the periphery of the gills (Figure 4A1). Given that fluorescence intensities in different animal gills varied strongly in epithelial cells, a ratio was calculated between fluorescence in endothelial and epithelial cells as explained in the section describing the fluorimetric nitric oxide analysis. The DAF-2T fluorescence increased more pronouncedly in endothelial than epithelial cells in a time and pO_2_ dependent manner (*K* = 50.983; *p* < 0.001, *n* = 4–9; Figures 4A2,B). The effect of hypoxia on NO formation was much greater (roughly 2 times more fluorescence) at 1 than at 7 kPa pO_2_. Compared to normoxic control conditions (Figure 4C1), treatment with SpNONOate caused DAF-2T fluorescence intensity to increase significantly at 30 min of exposure to the NO donor (Figures 4C2,D) (*K* = 20.877; *p* < 0.05, *n* = 3–5). No difference in DAF-fluorescence was observed between the effects of 3 mM and 6 mM SpNONOate in endothelial cells around the blood vessel (Figure 4D).

**Figure 5:**
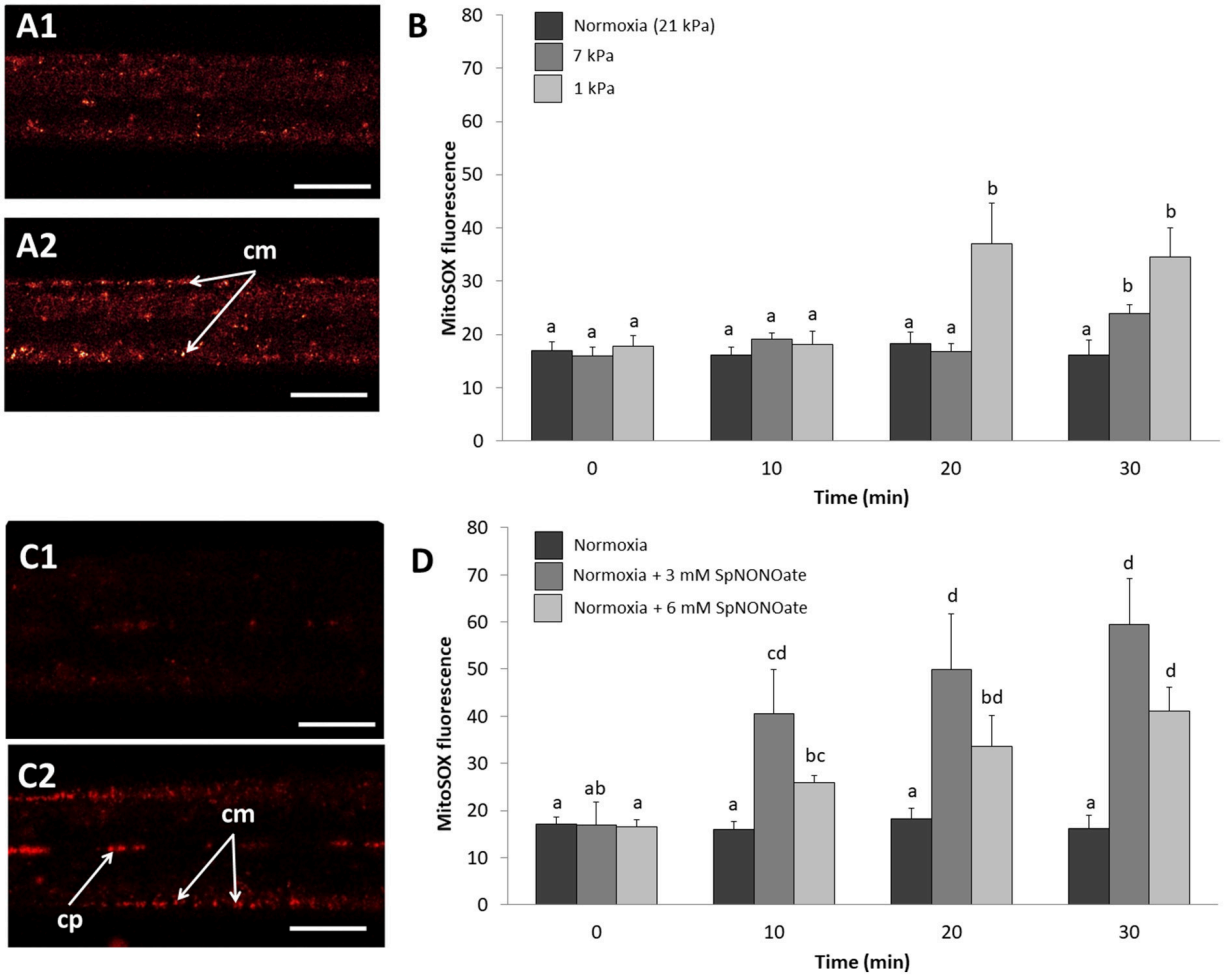
Mitochondrial superoxide anion formation imaged by MitoSOX fluorescence in *Mytilus edulis* gill tissues over time when exposed to different: **(A,B)** degrees of hypoxia or **(C,D)** concentrations of Spermine NONOate. **(A)** Representative images of the gills of the same individual with MitoSOX, **(A1)** taken under control (normoxic) conditions and **(A2)** under hypoxia 1 kPa for 30 min. **(C)** Representative images of the gills of the same individual under **(C1)** normoxia and **(C2)** incubated with 6 mM Spermine NONOate for 30 min. Quantitative data are shown in **(B,D)** and values are expressed as MitoSOX_OR_ – MitoSOX_IR_ (see Materials and Methods sections for details). Letters indicate significant differences between mean values based on Kruskal-Wallis test followed by U Mann-Whitney pairwise comparisons **(B)** or a one-way ANOVA followed by a Student-Newman-Keuls *post hoc* multiple comparison test **(D)**. Scale bars: 20 μm; cm, ciliary-associated mitochondria; cp, circulating particles.

Hypoxic exposure of gills caused a pO_2_ and time-dependent increase of ${\text{O}}_{2}^{∙-}$ formation in the mitochondria (MitoSOX fluorescence) within the epithelial cells of the outer gill region (Figures 5A1,2). In fact, MitoSOX fluorescence increased stepwise with hypoxic intensity (1 kPa > 7 kPa) with the tiered effect reaching statistical significance after 20 (1 kPa) and 30 min (7 kPa) (Figure 5B) (*K* = 33.532; *p* < 0.001, *n* = 4–6). Fluorescence intensity after application of SpNONOate under normoxia increased mainly in the mitochondria at the basis of the cilia themselves and thus in the outer periphery of the filaments (Figure 5C). The effect was significant for both the 3 mM (*F* = 3.352; *p* = 0.05, *n* = 3–5) and 6 mM (*F* = 5.762; *p* = 0.009, *n* = 3–5) SpNONOate treatments (Figure 5D). Furthermore, we observed that circulating particles/hemocyte cells in the hemolympahtic blood vessel emitted intense MitoSOX fluorescence under all experimental conditions including normoxia (Figure 5C).

The Δψm of cilia-associated (outer) mitochondria (JC-10 fluorescence ratio) remained unaffected over time of exposure to hypoxia or NO donor (21 kPa: *F* = 0.155; *p* = 0.925, *n* = 5; 7 kPa: *F* = 0.324, *n* = 5; *p* = 0.808; 1 kPa: *F* = 1.152; *p* = 0.357, *n* = 6; 3 mM SpNONOate: *F* = 0.520; *p* = 0.674, *n* = 5; 6 mM SpNONOate: *F* = 0.018; *p* = 0.996, *n* = 6) (Table 2). Likewise, no change of Δψm was observed in the mitochondria around the blood vessel (endothelial mitochondria) (21 kPa: *F* = 0.134; *p* = 0.938; 7 kPa: *F* = 0.761; *p* = 0.531; 1 kPa: *F* = 0.300; *p* = 0.825, *n* = 4–6; 3 mM SpNONOate: *F* = 0.013; *p* = 0.998, *n* = 5; 6 mM SpNONOate: *F* = 0.018; *p* = 0.997, *n* = 6) (Table 2).

**Table 2:** JC-10 ratio values (indicative of mitochondrial membrane potential) for inner (endothelial) and outer mitochondria (cilia-associated).

**Exposure time**	**0 min**		**10 min**		**20 min**		**30 min**	
**Treatment**	**Outer**	**Inner**	**Outer**	**Inner**	**Outer**	**Inner**	**Outer**	**Inner**
Normoxia (21 kPa)	0.70 ± 0.08	0.63 ± 0.07	0.62 ± 0.10	0.57 ± 0.09	0.69 ± 0.07	0.63 ± 0.08	0.68 ± 0.10	0.62 ± 0.09
7 kPa	0.99 ± 0.04	0.87 ± 0.03	0.98 ± 0.05	0.85 ± 0.04	1.05 ± 0.05	0.93 ± 0.04	1.01 ± 0.05	0.86 ± 0.04
1 kPa	0.82 ± 0.04	0.77 ± 0.04	0.77 ± 0.04	0.75 ± 0.03	0.76 ± 0.05	0.75 ± 0.04	0.70 ± 0.04	0.73 ± 0.03
Normoxia + 3 mM SpNONOate	0.86 ± 0.07	0.67 ± 0.09	0.83 ± 0.08	0.68 ± 0.07	0.77 ± 0.09	0.67 ± 0.08	0.70 ± 0.10	0.66 ± 0.08
Normoxia + 6 mM SpNONOate	0.7 ± 0.1	0.6 ± 0.1	0.7 ± 0.1	0.6 ± 0.1	0.6 ± 0.1	0.6 ± 0.1	0.7 ± 0.1	0.6 ± 0.1

Values are expressed as average ± SE.

**Figure 6:**
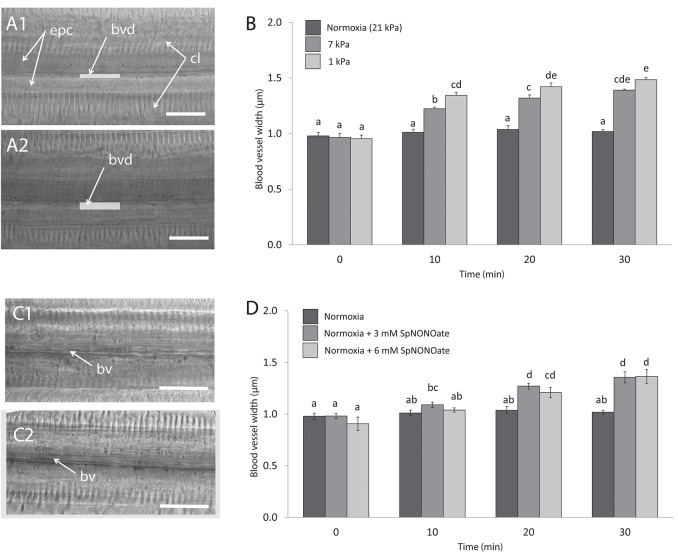
Blood vessel diameter in gill filaments exposed to different: **(A,B)** degrees of hypoxia and **(C,D)** concentrations of Spermine NONOate. **(A)** Representative transmission images of the gills of the same individual **(A1)** taken under control (normoxic) conditions and **(A2)** under hypoxia 1 kPa for 30 min. **(C)** Representative images of the gills of the same individual under **(C1)** normoxia and **(C2)** incubated with 3 mM Spermine NONOate for 30 min. Letters indicate significant differences between mean values based on one-way ANOVA followed by a Student-Newman-Keuls *post-hoc* multiple comparison test. Scale bars: 20 μm; bv: blood vessel, bvd: blood vessel diameter, indicated with a gray bar, cl: gill cilia, epc: epithelial cells.

Blood vessels under undisturbed conditions (normoxia) had an average width of 0.99 ± 0.01 μm (*N* = 29). In each piece of gill we analyzed, exposure to hypoxia caused an increase of the blood vessel diameter (Figures 6A1,2) and the effect differed significantly depending on the level of hypoxic intensities the gills experienced (*F* = 56.673; *p* < 0.001, *n* = 4–7; Figure 6B). The opening occurred rapidly and a 1.4- and 1.6-fold increase in blood vessel diameter was recorded when tissues were exposed to 7 and 1 kPa for 30 min, respectively (Figure 6B). The difference amounts to roughly a doubling (7 kPa) and tripling (1 kPa) of vessel cross sectional width compared to normoxia. Exposing gills to SpNONOate (Figure 6C) resulted in a significant increase of blood vessel diameter (*F* = 37.153; *p* < 0.001, *n* = 5) between controls and both concentrations applied: 1.4- and 1.5-fold increase for 30 min exposure to 3 and 6 mM SpNONOate, respectively. Values corresponding to 30 min exposure to 3 mM SpNONOate did not differ significantly from those registered for 7 kPa pO_2_ (*F* = 0.921; *p* = 0.365; Figure 6D).

### Respiratory Complex Activities

**Figure 7:**
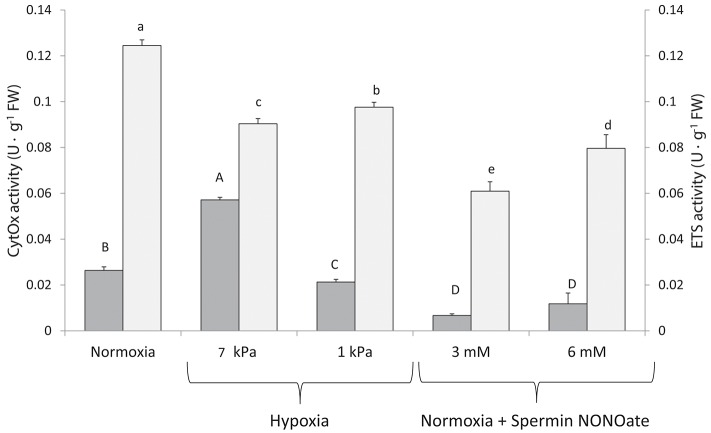
Cytochrome c oxidase (■) and electron transport system (□) activities recorded in excised gill tissues under different degrees of hypoxia and Spermine NONOate concentration. Different letters (capital and lowercase letters for cytochrome c oxidase and electron transport system, respectively) indicate significant differences between mean values based on U Mann-Whitney pairwise comparisons.

*In vitro* CytOx activity assayed in homogenates of frozen gill tissues (Figure 7: dark bars) increased significantly between the normoxic incubations (*n* = 12) and homogenates incubated at moderate hypoxia (7 kPa pO_2_, *K* = 42.499; *p* < 0.001, *n* = 12). The percentage increase when calculated for individual comparisons amounted to 125% of the activity in normoxia. Incubation of the same homogenates at severe O_2_ deficiency of 1 kPa pO_2_ reduced CytOx activity by 16% (*n* = 11 comparisons between normoxia and 1 kPa pO2, *F* = 8.338, *p* = 0.009). Addition of SpNONOate reduced CytOx activity on average by over 70% at 3 mM (*n* = 9, *F* = 101.502, *p* < 0.001) and by 35% at 6 mM (*n* = 9, *F* = 10.695, *p* = 0.004) compared to the normoxic controls without NO donor (Table S1).

Compared to normoxic conditions (*n* = 12), activity of both ETS complexes I and III in homogenates decreased at both hypoxic conditions (*n* = 12) and if assayed in the presence of SpNONOate (*n* = 10) (Figure 7, *K* = 41.993; *p* < 0.001). A decrease of 27 and 22% in ETS activity was recorded under moderate (*F* = 102.987, *p* < 0.001) and acute hypoxia (*F* = 73.929, *p* < 0.001), respectively. The addition of SpNONOate in normoxic medium caused a partial inhibition of the ETS activity by 51 and 34% for 3 mM (*K* = 13.635, *p* < 0.001) and 6 mM (*F* = 189.277, *p* < 0.001), respectively, based on mean values (Table S2).

## Discussion

The respiratory response of *M. edulis* to declining environmental O_2_ tension has been the focus of a great number of studies over the past 50 years, as researchers became fascinated with the enormous physiological flexibility of this successful colonizer of marine coastal habitats (Zandee et al., 1980) but also worried about the increasing impact of climate global change, mussel fisheries, neobiota, and hypoxia on the marine coastal environments (Eriksson et al., 2010).

### Nitric Oxide: A Local Vasodilator in *M. edulis* Endothelial Gill Cells

In the present paper we documented widening of the hemolymphatic vessel in isolated gill filaments exposed to hypoxia in a microscopic chamber. Increasing NO (DAF-2T) fluorescence in the endothelial muscle cells below 7 kPa is a strong indication that NO functions as a hypoxic messenger and local vasodilator in these gills pieces. The effect is rapid and sets on after 10 min of filament exposure; and both effects, NO accumulation and relaxation of the muscular endothelium that causes opening of the blood vessel increase in parallel as pO_2_ declines to 1 kPa. A 50% increase of the blood vessel lumen under hypoxia appears as a rather strong effect detected with our approach. By contrast, experimental exposure of gill pieces to addition of NO donor had less pronounced effects on both parameters, presumably because the addition occurred in normoxic medium in which NO is rapidly oxidized to ${\text{NO}}_{2}^{-}$. Still, also in this experiment, 50% increase of the blood vessel lumen was observed after 20 and 30 min in NO donor treated samples, but not in control gills maintained in the chamber for the same duration under normoxic conditions. This experiment supports the notion that NO acts as mediator of blood vessel relaxation in *Mytilus*. The experiment with SpNONOate followed a different dynamic because the NO donor was added to the outside medium and NO accumulated only in the endothelial cells of the blood vessel. NO diffuses freely through membranes and can reach the endothelium from the outside within seconds, especially as heme-containing blood pigments are absent in *Mytilus*. Alternatively, NO with a vasodilatory effect could also be released from the strongly DAF-2T fluorescing hemocyte cells moving within the blood vessel (Rivera-Ingraham et al., 2016), which play a role in bivalve immune response (Philipp et al., 2012). NO emitted within the blood vessel could also function as blood-borne vasodilator, triggered under hypoxic conditions or during parasite invasion (Kaiser et al., 1989, 1991).

At any rate this is—to our knowledge—the first explicit report of a vasodilatory effect of NO in endothelial cells of mussel gills. Biological functions of NO have been reported for terrestrial and aquatic non-model invertebrates (summarized in Palumbo, 2005), but its involvement in regulating blood pressure is so far documented only in cephalopods by Schipp and Gebauer (1999). Our study underlines NO induced vasodilation to be an evolutionarily old mechanism of endothelial cells (Jacklet, 1997). Indeed it is logical to reduce vessel resistance when heart beat increases in a hypoxic mussel (Bayne, 1971).

### Effects of Hypoxia and Nitric Oxide Donor on Gill Respiration, Mitochondria, and Respiratory Chain Components

Experimental addition of NO donor to freshly excised gill pieces inhibited gill respiration in a concentration and pO_2_ dependent manner. Indeed, addition of 6 mM SpNONOate completely abolished respiration under near anoxic conditions. The pO_2_ dependency of the inhibition effect is a clear indication of NO oxidation in the normoxic medium. Even at 21 kPa pO_2_, 6 mM SpNONOate decreased respiration by 44% compared to NO free controls directly at the start of experimentation. Data on NO concentrations in bivalve shell water or hemolymph are not available so far, but NO concentrations that cause blood vessel relaxation in mammals are in the low nM range (Demoncheaux et al., 2002), suggesting that sufficient amount of NO was generated in our SpNONOate experiments to achieve a vasodilatory effect. This is a hint that NO accumulating in shell water and hemolymph as O_2_ diminishes during shell closure might also have a physiological effect on gill performance and potentially reduces metabolic rate also in other tissues.

The NO donor treatment of cell free gill homogenates during 3 h strongly inhibited both CytOx (complex IV) and ETS (complex I and III) activities in a concentration independent manner, which suggests inhibition of respiratory complexes to be saturated at 3 mM SpNONOate. These assays in which we apparently applied excess amounts of NO donor were intended to clarify chemical susceptibility of *M. edulis* respiratory complexes to NO inhibition. This being confirmed, it seems likely that the strong effect of NO donor administration on respiration of freshly excised intact gill pieces is due to the inhibition of the mitochondrial electron transport complexes. The multisite inhibitory effect is known from experiments with rat heart submitochondrial particles (SMPs) that were exposed to slightly higher concentrations of NO (0.1 and 0.3 μM, Poderoso et al., 1996). The authors found substantial inhibition of NADH-cytochrome c reductase (complex I), as well as complex III (ubiquinone-cytochrome b reductase) with the strongest inhibition of complex IV (CytOx, 50% inhibition with 0.1 μM NO). Higher effective concentrations of NO (0.3–0.6 μM) caused significant H_2_O_2_ release from SMPs supplied with succinate as respiratory electron donor, indicating ${\text{O}}_{2}^{∙-}$ production, which in aqueous media rapidly dismutates to H_2_O_2_. In intact tissues, ${\text{O}}_{2}^{∙-}$ produced by NO inhibited mitochondria would rapidly form peroxynitrite (ONOO^−^) and cause oxidative and cellular damage at NO concentrations that, according to these authors, are about one order of magnitude higher than the concentrations affording inhibition of CytOx (Poderoso et al., 1996).

Hypoxia had a strong effect on respiration rates and the activities of respiratory chain complexes I, III, and IV. It is interesting to note that CytOx activity measured in the homogenates incubated at mildly hypoxic pO_2_ (7 kPa) was higher than in homogenates from the same gill assayed after normoxic incubation (21 kPa). This would have appeared as an artifact, if not replicated with 12 individual gill experiments, and if the activity maximum at 7 kPa pO_2_ had not been observed in the range of the elevated respiration pattern shown for *M. edulis* gills in our previous study (Rivera-Ingraham et al., 2013b). As all gills originated from the same batch of control mussels taken directly from the holding tank, physiological differences in the extracts incubated at different pO_2_ levels can be excluded. It appears that in the 21 kPa O_2_ treatment something may have inhibited CytOx activity, such as a potential production of ROS in the air equilibrated homogenates. Indeed, mitochondrial ROS formation has been shown to increase linearly with O_2_ concentration (Turrens et al., 1982) and studies in invertebrates have shown that ROS formation is higher under normoxia than under reduced O_2_ conditions (e.g., Rivera-Ingraham et al., 2013a). As plenty of O_2_ was present in the normoxic homogenate based assay that was not reduced by CytOx activity, it may very well have caused ${\text{O}}_{2}^{∙-}$ formation from autoxidation of ETS compounds as shown for hyperoxically perfused rat lung endothelia (Brueckl et al., 2006) or from other cellular compounds easily prone to oxidation once exposed to air. Hence the results obtained with the cell free homogenates must not be over interpreted in terms of their physiological implications for the intact tissue.

It was also interesting to see that ${\text{O}}_{2}^{∙-}$ formed in the mitochondria of the intact gill filaments exposed to hypoxia and near anoxia, and that ${\text{O}}_{2}^{∙-}$ production was even more strongly induced by NO donor treatment in peripheral than in endothelial mitochondria (Figure 5C2). Thus, ${\text{O}}_{2}^{∙-}$ production appears to be induced as mitochondrial electron transport slows near anoxia and can also be triggered by NO as a mitochondrial ETS inhibitor in *M. edulis*. The effect of the NO donor was not only stronger but also quicker, and could potentially mimic an oxidative burst reaction triggered by NO. It is characteristic of marine invertebrate mitochondria that their lower membrane potential and flexibly adjustable proton leak supports mitochondrial integrity and stabilizes inner Δψm against environmental factor fluctuations including hypoxia (Abele et al., 2007). Stable Δψm in epithelial and endothelial gill mitochondria, recorded with the membrane potential sensitive fluorophore JC10 in our short-term hypoxia and SpNONOate exposure experiments, underline this mitochondrial tolerance. It might have been different if experimental exposure of gill pieces had persisted over longer time than 30 min. In meiofauna flatworms we observed an increase of Δψm with life imaging following 1.5 h in anoxia (Rivera-Ingraham et al., 2013a). A potential mechanism to stabilize Δψm is the alternative oxidase (AOX) that enables branching electron transport and maintenance of Δψ_m_ when CytOx becomes inhibited by cyanide, hydrogen sulfide (Parrino et al., 2000), or NO (further reading in Abele et al., 2017). Recently AOX sequences have been reported from several molluscan species, among them *Mytilus californianus* and *M. galloprovincialis* (McDonald et al., 2009). This is a very powerful and efficient mechanism in hypoxia tolerant aquatic invertebrates, and it seems likely that AOX may have stabilized Δψm against short term hypoxic exposure in *M. edulis* gills.

## Conclusion and Future Perspectives

With this study we highlighted for the first time that NO, formed in the endothelial muscle cells around the hemolymphatic vessel of *M. edulis* gill filaments, can function as a local vasodilator under hypoxic conditions. Externally applied NO has a concentration dependent effect on gill respiration and blood vessel diameter and might affect the physiological performance of the gills and other tissues if it accumulates to significant amounts in the nM range under hypoxic conditions. Indeed, shell water NO and potentially ${\text{NO}}_{2}^{-}$ levels [see (van Faassen et al., 2009) for effects of ${\text{NO}}_{2}^{-}$ on cellular signaling and mitochondrial respiration under hypoxic conditions] could have a mediating effect inducing metabolic shut down in hypoxia-exposed or stress exposed mussels that keep the shell closed for prolonged periods. A controlled metabolic shut down and a tiered reduction of ETS activities, including CytOx, may prevent significant ROS formation during hypoxic and anoxic transgression. All this may show an ancient mechanism for controlling respiratory electron transport under conditions of variable environmental oxygenation, typical for coastal marine environments to date. The interaction between animals and their microbial biofilms is a fascinating future topic that could also be of relevance with respect to hypoxic adaptation of marine benthic macrofauna.

## Author Contributions

All co-authors planned the experiments together and designed the workplan. PG and GR-I carried out the main body of experimental work in the laboratory of DA. GR-I, PG, and DA analyzed the data and produced the figures and tables. All authors contributed to the discussion and the writing of the manuscript.

### Conflict of Interest Statement

The authors declare that the research was conducted in the absence of any commercial or financial relationships that could be construed as a potential conflict of interest.
