# Supplementary material for: Hypoxically Induced Nitric Oxide: Potential Role as a Vasodilator in Mytilus edulis Gills
Source: Front Physiol. 2019 Mar 5;9:1709. doi: 10.3389/fphys.2018.01709 (PMC6411825; doi:10.3389/fphys.2018.01709)
Supplement: Supplementary file 1 [file Table_1.docx]

**Table S1:** Cytochrome c oxidase (CytOx) activity expressed as U CytOx · g fresh weight ^-1^ for each of the treatments considered. Values in brackets represent the positive or negative differences in activity in percent compared to normoxic values

| **Ind** | **Normoxia** | **7 kPa** | **1 kPa** | **Normoxia + 3 mM SpNONOate** | **Normoxia + 6 mM SpNONOate** |
| --- | --- | --- | --- | --- | --- |
| 1 | 0.027 | 0.057 (113) | 0.024 (-10) |  |  |
| 2 | 0.039 | 0.063 (64) | 0.022 (-44) | 0.008 (-79) | 0.005 (-87) |
| 3 | 0.026 | 0.055 (115) |  |  |  |
| 4 | 0.027 | 0.054 (105) | 0.020 (-26) | 0.005 (-81) | 0.005 (-83) |
| 5 | 0.028 | 0.054 (91) | 0.020 (-28) | 0.002 (-94) | 0.003 (-88) |
| 6 | 0.024 | 0.055 (136) | 0.019 (-21) | 0.017 (-27) | 0.005 (-79) |
| 7 | 0.020 | 0.064 (222) | 0.023 (17) |  |  |
| 8 | 0.027 | 0.058 (114) | 0.023 (-16) | 0.003 (-88) | 0.007 (-73) |
| 9 | 0.017 | 0.052 (202) | 0.021 (20) | 0.013 (-25) | 0.008 (-56) |
| 10 | 0.024 | 0.060 (149) | 0.019 (-20) | 0.003 (-89) | 0.010 (-59) |
| 11 | 0.032 | 0.057 (79) | 0.019 (-40) | 0.008 (-76) | 0.008 (-74) |
| 12 | 0.026 | 0.054 (110) | 0.024 (-8) | 0.047 (82) | 0.009 (-65) |
