# Supplementary material for: Hypoxically Induced Nitric Oxide: Potential Role as a Vasodilator in Mytilus edulis Gills
Source: Front Physiol. 2019 Mar 5;9:1709. doi: 10.3389/fphys.2018.01709 (PMC6411825; doi:10.3389/fphys.2018.01709)
Supplement: Supplementary file 2 [file Table_2.docx]

**Table S2:** Electron transport system (ETS) activity expressed as U ETS · g fresh weight ^-1^ for each of the treatments considered. Values in brackets represent the percentage over normoxic values

| **Ind** | **Normoxia** | **7 kPa** | **1 kPa** | **Normoxia + 3 mM SpNONOate** | **Normoxia + 6 mM SpNONOate** |
| --- | --- | --- | --- | --- | --- |
| 1 | 0.137 | 0.099 (-27.919) | 0.105 (-23.607) |  |  |
| 2 | 0.138 | 0.089 (-35.535) | 0.098 (-28.476) | 0.118 (-13.868) | 0.086 (-37.529) |
| 3 | 0.115 | 0.084 (-27.226) |  |  |  |
| 4 | 0.125 | 0.085 (-32.150) | 0.099 (-20.611) | 0.058 (-53.546) | 0.065 (-48.052) |
| 5 | 0.128 | 0.087 (-31.440) | 0.099 (-22.121) | 0.098 (-23.015) | 0.077 (-39.896) |
| 6 | 0.130 | 0.098 (-24.963) | 0.102 (-22.130) | 0.094 (-28.044) | 0.078 (-40.002) |
| 7 | 0.118 | 0.083 (-29.744) | 0.091 (-22.852) |  |  |
| 8 | 0.121 | 0.085 (-29.663) | 0.098 (-19.420) | 0.072 (-40.941) | 0.069 (-43.083) |
| 9 | 0.115 | 0.084 (-26.967) | 0.095 (-17.513) | 0.062 (-46.507) | 0.060 (-47.780) |
| 10 | 0.119 | 0.082 (-30.830) | 0.090 (-24.632) | 0.063 (-46.890) | 0.062 (-48.211) |
| 11 | 0.133 | 0.092 (-31.179) | 0.108 (-18.528) | 0.081 (-38.949) | 0.055 (-58.448) |
| 12 | 0.114 | 0.067 (-40.882) | 0.088 (-23.286) | 0.049 (-56.975) | 0.033 (-71.348) |
